# MHC-Matched Induced Pluripotent Stem Cells Can Attenuate Cellular and Humoral Immune Responses but Are Still Susceptible to Innate Immunity in Pigs

**DOI:** 10.1371/journal.pone.0098319

**Published:** 2014-06-13

**Authors:** Yoshihisa Mizukami, Tomoyuki Abe, Hiroaki Shibata, Yukitoshi Makimura, Shuh-hei Fujishiro, Kimihide Yanase, Shuji Hishikawa, Eiji Kobayashi, Yutaka Hanazono

**Affiliations:** 1 Division of Regenerative Medicine, Center for Molecular Medicine, Jichi Medical University, Tochigi, Japan; 2 Center for Development of Advanced Medical Technology, Jichi Medical University, Tochigi, Japan; 3 Tsukuba Primate Research Center, National Institute of Biomedical Innovation, Ibaraki, Japan; 4 CREST, Japan Science and Technology Agency, Tokyo, Japan; Beth Israel Deaconess Medical Center, Harvard Medical School, United States of America

## Abstract

Recent studies have revealed negligible immunogenicity of induced pluripotent stem (iPS) cells in syngeneic mice and in autologous monkeys. Therefore, human iPS cells would not elicit immune responses in the autologous setting. However, given that human leukocyte antigen (HLA)-matched allogeneic iPS cells would likely be used for medical applications, a more faithful model system is needed to reflect HLA-matched allogeneic settings. Here we examined whether iPS cells induce immune responses in the swine leukocyte antigen (SLA)-matched setting. iPS cells were generated from the SLA-defined C1 strain of Clawn miniature swine, which were confirmed to develop teratomas in mice, and transplanted into the testes (*n* = 4) and ovary (*n* = 1) of C1 pigs. No teratomas were found in pigs on 47 to 125 days after transplantation. A Mixed lymphocyte reaction revealed that T-cell responses to the transplanted MHC-matched (C1) iPS cells were significantly lower compared to allogeneic cells. The humoral immune responses were also attenuated in the C1-to-C1 setting. More importantly, even MHC-matched iPS cells were susceptible to innate immunity, NK cells and serum complement. iPS cells lacked the expression of SLA class I and sialic acids. The in vitro cytotoxic assay showed that C1 iPS cells were targeted by NK cells and serum complement of C1. In vivo, the C1 iPS cells developed larger teratomas in NK-deficient NOG (T-B-NK-) mice (*n* = 10) than in NK-competent NOD/SCID (T-B-NK+) mice (*n* = 8) (*p*<0.01). In addition, C1 iPS cell failed to form teratomas after incubation with the porcine complement-active serum. Taken together, MHC-matched iPS cells can attenuate cellular and humoral immune responses, but still susceptible to innate immunity in pigs.

## Introduction

Induced pluripotent stem (iPS) cells are generated from adult mature cells following transduction with the transcription factors *Oct3/4*, *Sox2*, *Klf4*, and *c-Myc* [1], [2]. They have the ability to differentiate into all cell types [3], [4], [5] and, therefore, have considerable potential for autologous stem cell therapies [6], [7]. Recently, it was shown that iPS cells do not elicit immune responses when they are transplanted into syngeneic mice [8], [9] or into autologous monkeys [10], [11]. Therefore, it is likely that human iPS cells would not elicit immune responses in an autologous setting [12].

However, autologous derivation of iPS cells requires considerable time and effort for their generation and for assessment of their clinical safety and efficacy. Therefore, the establishment of human leukocyte antigen (HLA)-typed banks of iPS cells for cell transplantation therapies has been proposed [13], [14], [15], [16]. Given that such banks would involve HLA-matched allogeneic human iPS cells being used for medical applications, a better model system is needed to reflect the likely HLA-matched settings and testing and developing medical applications. As swine show close similarities in anatomy, biochemistry and physiology to humans, they have been used as a possible model system [17], [18]. In addition, swine are the only large animal that have inbred MHC-defined strains available [19], [20], [21]. One such strain, Clawn miniature swine, was established in 1978 by Nakanishi and colleagues at Kagoshima University, and its swine leukocyte antigen (SLA) genotype was identified [20], [22], [23]. Previously, we established iPS cells from inbred SLA-defined Clawn miniature swine [24] (Fig. S1 in File S1). The transplantation of porcine iPS cells in an SLA-matched setting might provide a robust model for transplantation of human iPS cells in an HLA-matched setting. In the present study, we sought to determine whether iPS cells from Clawn miniature swine induce immune responses in an SLA-matched setting.

## Materials and Methods

### Animals

Clawn miniature swine (7- to 20-month-old male and female pigs) were purchased from Japan Farm (Kagoshima, Japan). Clawn miniature swine have two SLA haplotypes, C1 and C2 [22], [23]. As shown in the genealogy (Fig. S2 in File S1), the donor animal (AT25) and the recipients (CT19, CQ38, CU65, SF65, SD57 and CQ74) were very closely related. All pigs were housed at 23–27°C under conditions of 50–70% humidity with a 12 hours light/12 hours dark cycle. All animals were housed in individual pens (1 m×1.5 m) with a bedding space (0.5 m×1 m). They were given 600 g of feed once daily.

Six-week-old non-obese diabetic/severe combined immunodeficient (NOD/SCID) and NOD/SCID/γc^null^ (NOG) male mice were purchased from CLEA Japan (Tokyo, Japan) and the Central Institute for Experimental Animals (Kawasaki, Japan), respectively. The mice were maintained under a 12 hours light/12 hours dark cycle and fed ad libitum.

All animal experiments reported here were approved by the Institutional Animal Experiment Committee of Jichi Medical University (mice 13490 and pigs 13450).

### Porcine iPS cells

C1 iPS cells were established from porcine embryonic fibroblasts (PEFs) of a C1 strain animal using retroviral vectors expressing human SOX2, OCT3/4, KLF4, and c-MYC [24]. The cells were maintained on a feeder layer of mitomycin C (Kyowa-Hakko-Kogyo, Tokyo, Japan)-treated STO cells (ATCC, VA, USA) in Knockout DMEM (Invitrogen, CA, USA) supplemented with 15% fetal bovine serum (Invitrogen), 1% glutamax-L (Invitrogen), 1% nonessential amino acids (Invitrogen), 0.1 mM 2-mercaptoethanol (Invitrogen), 50 U/ml penicillin (Invitrogen), 50 µg/mL streptomycin (Invitrogen), 10 µM forskolin (Biomol International Inc), and in-house produced recombinant porcine leukemia inhibitory factor (1∶200 of conditioned medium) at 38.5°C in a humidified atmosphere of 5% CO_2_ in air.

### Transplantation

Transplantation experiments were performed in the Center for Development of Advanced Medical Technology of Jichi Medical University and in the Japan Farm Clawn Institute. For transplantation into pigs, C1 iPS cells were harvested by trypsinization and then incubated on gelatin-coated dishes for 15 min to remove STO feeder cells. Harvested iPS cells were washed by centrifugation and suspended in 500 µL of phosphate-buffered saline (PBS). Recipient pigs were anesthetized by 5% isoflurane inhalation. The cells were injected (3×10^7^ cells/site) into the testes of the C1 pigs (*n* = 4) using 23-gauge needles (Terumo, Tokyo, Japan) in combination with ultrasound guidance (Fig. S2 in File S1). C1 iPS cells were also injected into the ovaries of C1 (*n* = 1) and C2 (*n* = 1) pigs after laparotomy (Fig. S2 in File S1). At 47 or 125 days after injection, the pigs were euthanized by administration of KCl solution under general anesthesia.

For transplantation into mice, C1 iPS cells were injected (1×10^6^ cells/site) intramuscularly into the hindlimb of NOD/SCID and NOG mice using 29-gauge needles (Terumo). At 5 or 16 weeks after transplantation, the mice were euthanized and tumors were isolated.

### Immunohistochemistry

Tissue samples were fixed with 4% paraformaldehyde in PBS (Wako, Osaka, Japan) and embedded in paraffin. For immunostaining, the sections were first blocked using 2% bovine serum albumin (Sigma-Aldrich, MO, USA) in PBS and then stained with anti-porcine CD3 (Abcam, MA, USA; 1∶50), and anti-human CD79 (Dako, CA, USA; 1∶50). Some sections were stained with hematoxylin and eosin.

### Cytochemical staining

For immunocytochemistry, cells were fixed with 4% paraformaldehyde in PBS and permeabilized with 0.2% Triton X-100 in PBS (Sigma-Aldrich). The cells were then blocked with 2% bovine serum albumin in PBS and incubated with primary antibodies. The cells were washed and then incubated with fluorescence-labeled secondary antibodies and 1 µg/ml DAPI (Roche Applied Science, USA; 1∶1000) to stain the nuclei. The primary antibodies used were antibodies against Oct3/4 (Santa Cruz Biotechnology, TX, USA; 1∶200), SLA class I (Vmrd, WA, USA; 1∶100), and CD47 (Thermo Fisher Scientific, CA, USA; 1∶50). The secondary antibodies used were anti-mouse or anti-rabbit IgG conjugated with FITC (Invitrogen, 1∶500) or Alexa Flour 555 (Invitrogen, 1∶500).

For lectin cytochemistry, cells were incubated with biotinylated *Maackia amurensis lectin* II (MAL, Vector Laboratories, USA; 1∶25) or FITC-conjugated *Sambucus nigra lectin* (SNA, Vector Laboratories, USA; 1∶40) at 4°C overnight. FITC-ultravidin (Leinco Technologies, MO, USA; 1∶200) was applied to the MAL-treated cells for 1 hour at room temperature.

### Mixed lymphocyte reaction (MLR)

Peripheral blood mononuclear cells (PBMCs) were isolated from porcine peripheral blood using Ficoll-Paque PLUS (GE Healthcare, Buckinghamshire, UK) following the manufacturer's procedures. PBMCs from SLA-matched recipients (C1) were suspended in RPMI-1640 (Gibco) medium with 10% FBS as responder cells. Then, 1×10^5^ responder cells and 2×10^4^ mitomycin C-treated stimulator cells were plated in each well of 96-well U-bottomed plates (Becton Dickinson, USA) and incubated at 38.5°C for 5 days. Plates were pulsed with 1 µCi/well of ^3^H-thymidine (GE Healthcare) for 24 hours and the cellular uptake of ^3^H-thymidine was quantified using a β-scintillation counter (Aloka, Tokyo, Japan). Stimulation index were represented by the mean of cpm experimental/cpm unstimulated. Significant differences were examined using Student's *t*-test.

### Humoral immune response

The presence of porcine IgG antibodies against C1 iPS cells in pigs was determined using a flow cytometer. C1 iPS cells were incubated with 1∶10 diluted serum taken from the C1 iPS cell-transplanted C1 pigs, and from allogeneic pigs that were not C1 or C2. After secondary staining with FITC-conjugated anti-porcine IgG specific for Fc fragment (AbD Serotec, Oxford, UK), the cells were examined by FACS Calibur flow cytometer. Data acquisition and analysis were performed using CellQuest software (BD Pharmingen).

### In vitro NK-cell mediated cytotoxicity assay

PBMCs were isolated by density gradient centrifugation using Ficoll-Paque PLUS to exclude polymorphonuclear cells. The cells were incubated with BD Pharm Lyse Buffer (BD Bioscience) to remove erythrocytes. The cells were labeled with a PE-conjugated anti-porcine CD16 antibody (AbD Serotec). The labeled cells were isolated with anti-PE paramagnetic microbeads (Miltenyi Biotec, Bergisch-Gladbach, Germany) according to the manufacture's instructions. The isolated cells were labeled with an FITC-conjugated anti-porcine CD3 antibody (BD Bioscience). Using a Cell Sorter SH800 (SONY, Tokyo, Japan), the fraction of CD3-negative and CD16-positive cells was collected. The collected cells were used as porcine NK cells. C1 iPS cells were incubated as target cells with NK cells at ratios of 1∶30 for 6 h at 38.5°C in DMEM supplemented with 1% FBS, 0.1 mM 2-mercaptoethanol and 1 ng/ml recombinant porcine IL-2 (R&D systems). The cytotoxicity of C1 iPS cells to NK cells was assessed by lactate dehydrogenase (LDH) release assay. Percent cytotoxicity was calculated as follows: cytotoxicity (%) = (Experimental value – Effector control – Target control)×100/(High control – Target control). The High control was obtained after incubating C1 iPS cells with 2% Triton X-100. The Effector control and the Target control were obtained after incubating NK cells alone and C1 iPS cells alone, respectively.

### In vitro complement-mediated cytotoxicity assay

The cytotoxicity of C1 iPS cells to serum complement was assessed by LDH release assay. C1 iPS cells were incubated with C1 porcine serum that was heat–non-treated (complement–non-inactivated) at ratios of 3∶100 to 30∶100 for 30 min at 38.5°C. The supernatants were centrifuged at 250× *g* for 10 min and examined for the release of LDH using the Cytotoxicity Detection Kit (Takara Bio Inc, Tokyo, Japan). Percent cytotoxicity was calculated as follows: cytotoxicity (%) = (Experimental value – Low control)×100/(High control – Low control). Low and High controls were obtained after incubating C1 iPS cells alone or with 2% Triton X-100, respectively.

### In vitro phagocytosis assay

PBMCs were plated at 5×10^4^ cells per well in a 24-well tissue-culture plate at 38.5°C for 2 hours to allow peritoneal macrophages to attach to the plate. After washing off the non-adherent cells, 2×10^5^ EGFP-labeled C1 iPS cells were then added to each well as target cells. After co-incubation of macrophages and iPS cells with or without CD47-blocking antibody (BRIC126, Santa Cruz; 1∶50) for two hours, macrophages were stained with anti-porcine monocytes antibody (Antigenix America, USA; 1∶100). Phagocytic index (PI) was calculated as the number of engulfed iPS cells per 100 iPS cells.

### Reverse transcription-polymerase chain reaction (RT-PCR)

Reverse transcription reactions were performed using a Thermo Scientific Verso cDNA Synthesis kit (Thermo) with random hexamer primers. PCR was performed with Ex-Taq (Takara Bio Inc). PCR products were separated on 2% agarose gel and visualized by ethidium bromide staining. Semi-quantitative RT-PCR was performed using an ABI Step One (Applied Biosystems, CA, USA) with SYBR green PCR mix (Qiagen, CA, USA). Primer sequences are listed in Table S1.

### Statistical analysis

Data are expressed as mean ± standard deviation. The significance of differences between groups was tested using Student's *t*-test.

## Results

### Cellular and humoral immune responses in an SLA-matched setting

First, we transplanted porcine iPS cells derived from the C1 strain of Clawn miniature swine [24] into immunodeficient mice, and confirmed formation of teratomas from C1 iPS cells (Fig. S1 in File S1). Next, we tried to develop teratomas in pigs. Pigs were treated with immunosuppressants (tacrolimus, mycophenolate mofetil and steroid). Within 1–2 months after administration, they suffered from severe infection such as mediastinitis and reached the end-point when they should be euthanized. Thus, it is technically and ethically difficult to maintain such pigs until transplanted iPS cells develop teratomas. Then, we transplanted C1 iPS cells (3×10^7^ cells/site) into the testes (*n* = 4) or ovary (*n* = 1) of SLA-matched swine (i.e., C1-to-C1) without immunosuppression (Fig. S2 in File S1). C1 iPS cells were also transplanted into the ovary (*n* = 1) of an SLA-mismatched C2 female pig (i.e., C1-to-C2) without immunosuppression. No teratomas developed in any of the recipients tested on days 47 to 125 after transplantation.

**Figure 1:**
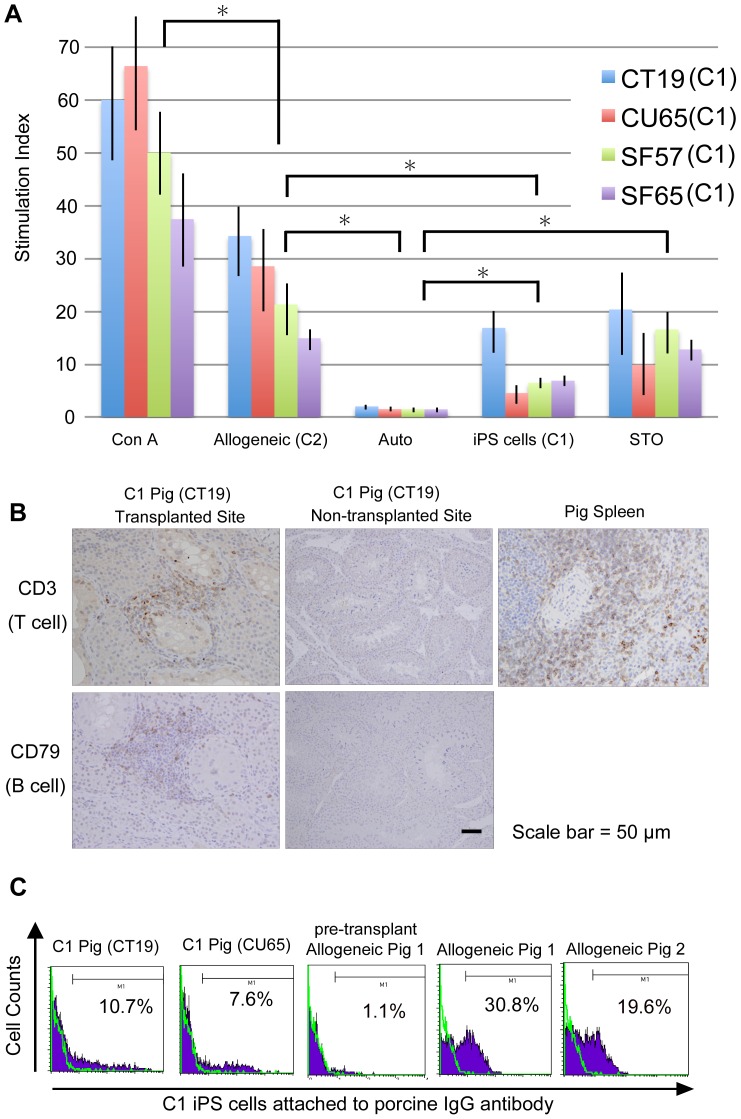
Cellular and humoral immune responses in an SLA-matched setting. (A) Mixed lymphocyte reactions (MLRs) against allogeneic (C2) porcine embryonic fibroblasts (PEFs), C1 iPS cells and STO feeder cells. Peripheral blood mononuclear cells (PBMCs) supplemented with concanavalin A (Con A) and C2 PEFs were used as positive controls, and autologous PBMCs (C1) were used as a negative control. The stimulation index of C1 iPS cells was significantly lower than that of allogeneic cells (^*^ *p*<0.01), but significantly higher than that of autologous cells (^*^ *p*<0.01). MLR was performed in triplicate and repeated three times and a typical result was shown. (B) Immunohistochemical staining with anti-CD3 and anti-CD79 antibodies. Pig spleen was stained with the anti-CD3 antibody as a positive control. Slight infiltration of CD3+ T-cells and CD79+ B cells was detected at the transplantation site in the SLA-matched pig CT19. (C) Porcine IgG antibodies against C1 iPS cells were determined by flow cytometry. As SLA-mismatched recipients, miniature pigs that were not C1 or C2 were used. The porcine IgG against C1 iPS cells was detected at much lower levels in the SLA-matched C1 pigs (CT19 and CU65) than in the SLA-mismatched allogeneic pigs. Cells labeled with secondary antibody without porcine serum as a negative control.

Mixed lymphocyte reaction (MLR) assays showed that the T-cell response to SLA-matched C1 iPS cells was indeed attenuated compared to SLA-mismatched C2 cells (Fig. 1A), but still stronger than to autologous cells. Notably, the T-cell reactivity of CT19 pig was higher compared to other recipients (Fig. 1A). This particular recipient showed in vivo evidence of inflammation and infiltration of a few CD3+ T cells and CD79+ B cells at the transplanted site (Fig. 1B). In addition, we performed ELISA to detect IFN-γ in the porcine serum. Although serum IFN-γ was clearly detected in the SLA-mismatched setting (C1-to-C2), it was undetectable in the SLA-matched setting (C1-to-C1) (Fig. S3 in File S1).

With regard to humoral immune responses, IgG against C1 iPS cells was detected in SLA-matched recipient pigs at day 17 or 19 after transplantation, but the level of IgG was lower than that in SLA-unmatched recipient pigs (Fig. 1C). Thus, matching of SLA could also attenuate the humoral immune response. Taken together, both cellular and humoral immune responses would be attenuated due to the matching of SLA.

### Involvement of natural killer (NK) cells in rejection of C1 iPS cells in vivo

**Figure 2:**
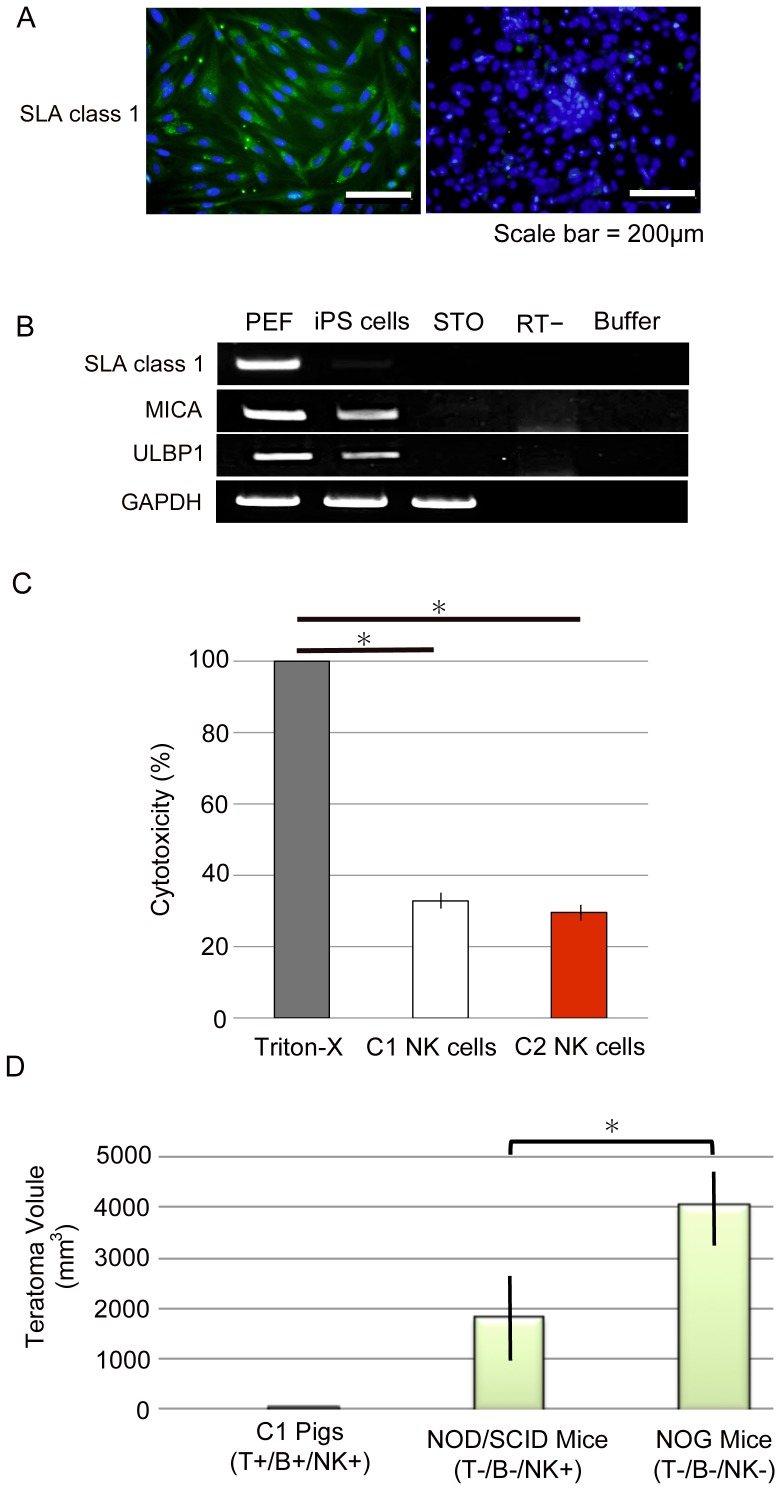
Susceptibility of C1 iPS cells to NK cells. (A) Immunocytochemical staining with an anti-porcine SLA class I antibody. Porcine PEFs showed positive staining for SLA class I, but C1 iPS cells were negative. (B) Reverse-transcription polymerase chain reaction (RT-PCR) analysis of the expression of *SLA class I* and ligands for NK cells. Lane 1, PEF; 2, C1 iPS cells; 3, STO feeder cells; 4, no reverse transcriptase; and 5, RT-PCR buffer alone. *MICA* and *ULBP1*, ligands for NK cells, were expressed on C1 iPS cells. *GAPDH* was used as a loading control. (C) Cell injury by NK cells was assessed by measuring LDH released in the supernatants as described in the Materials and Methods. Triton-X (2%) was used as a positive control. The percent cytotoxicity was quantified and shown as mean of triplicate. Two independent experiments were performed and similar results were obtained. (D) Estimated volumes of the teratomas in immunodeficient mice and SLA-matched pigs. C1 iPS cells were transplanted into NK-competent NOD/SCID (*n* = 14) and NK-deficient NOG (*n* = 14) mice, and C1 pigs (*n* = 5). After transplantation of C1 iPS cells (1×10^6^ cells/site) into immunodeficient mice, the teratomas were dissected and their size (diameter) was measured (^*^ *p*<0.01). After transplantation of C1 iPS cells (3×10^7^ cells/site) into C1 pigs, no teratomas were observed.

We then examined innate immune responses. C1 iPS cells express no or very low levels of SLA class I molecules [24] (Fig. 2A). Therefore, the injected C1 iPS cells are likely targets of NK cells, which kill MHC class I-negative cells [25]. In addition, our RT-PCR analysis demonstrated that C1 iPS cells expressed both MHC class I-related chain A (*MICA*) and UL16-binding protein 1 (*ULBP1*) (Fig. 2B). Previous reports demonstrated that pluripotent stem cells, such as ES cells, iPS cells, and germ-line stem cells are highly susceptible to NK cells because they express ligands for NK cells, such as *MICA* and *ULBP1* [26], [27]. The absence of SLA class I expression and the presence of NK ligands on C1 iPS cells suggest that they are susceptible to NK cells.

Therefore, we performed a cytotoxicity assay to examine whether porcine NK cells kill C1 iPS cells in vitro. As shown in Figure 2C, C1 iPS cells released LDH after incubation with even the SLA-matched NK cells, as well as the SLA-mismatched NK cells (Fig. 2C). This result indicates that C1 iPS cells are killed by NK cells. To examine whether the growth of C1 iPS cells are affected by NK cells in vivo, we transplanted 1×10^6^ C1 iPS cells/site intramuscularly into NK-competent NOD/SCID (T- B- NK+) mice and NK-deficient NOG (T- B- NK-) mice (Table 1). We found that the sizes of the teratomas that developed in NK-deficient NOG mice (*n* = 10) were significantly larger than those that developed in the NK-competent NOD/SCID mice (*n* = 8) (*p*<0.01) (Fig. 2D). These observations suggest that the overall growth of C1 iPS cells is hampered by NK cells in vivo.

**Table 1:** The teratoma formation in SLA-matched pigs and immunodeficient mice following injection of C1 iPS cells.

Recipients	SLA-matched pigs (T+/B+/NK+)	NOD/SCID mice (T-/B-/NK+)	NOG mice (T-/B-/NK−)	NOG mice injected with iPS cells cultured with serum-complement)
Number of teratoma formation per total	0/5	8/14	11/14	0/6
Frequency (%)	0	57.1	78.6	0

### Susceptibility of C1 iPS cells to complement-mediated cytotoxicity

**Figure 3:**
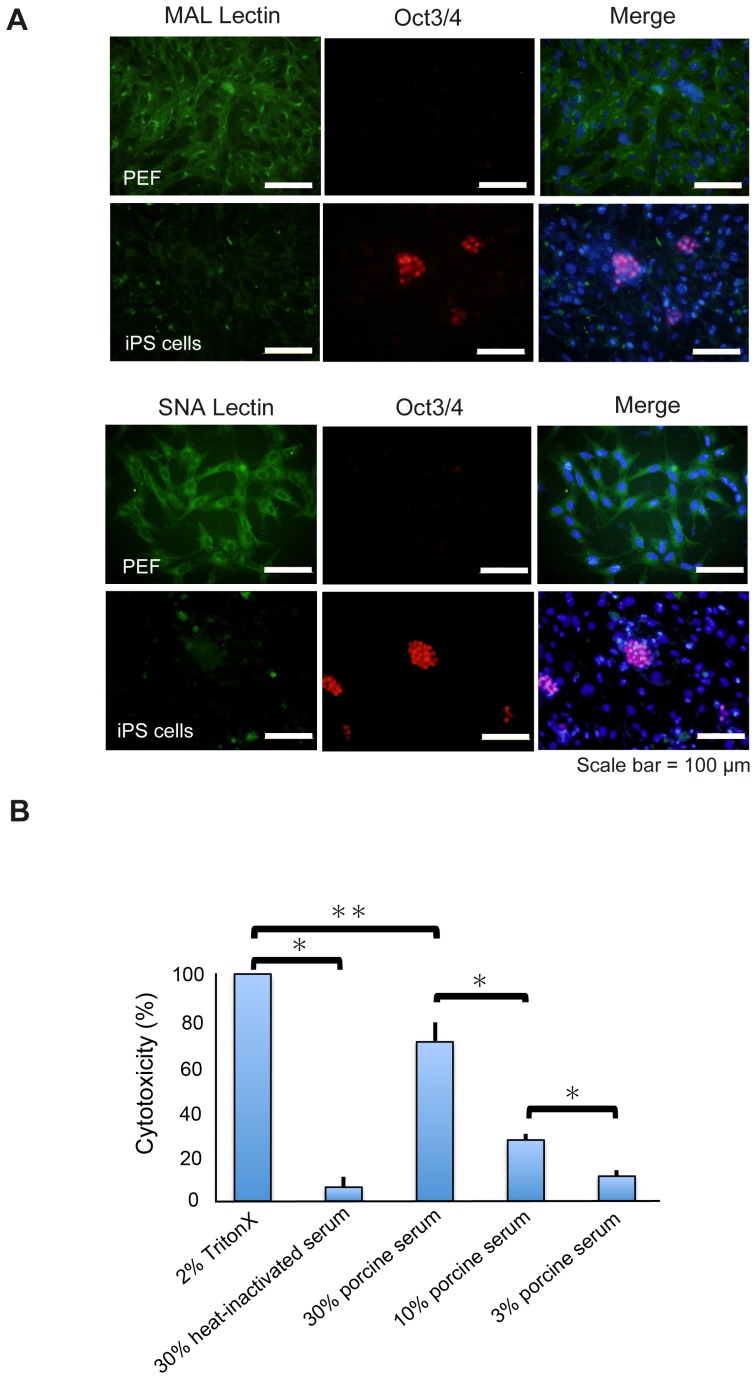
Complement-mediated cytotoxicity to C1 iPS cells. (A) Cytochemical assay for sialic acids using lectins. The expression of sialic acids was examined with SNA lectin and MAL lectin, both of which specifically bind to sialic acids. C1 iPS cells exhibited very low levels of sialic acids compared to fibroblasts. C1 iPS cells, but not PEFs, expressed Oct3/4. (B) Cell injury by complement was assessed by measuring LDH released in the supernatants described in the Materials and Methods. Triton-X (2%) was used as a positive control, and 30% of heat-inactivated (complement-inactivated) serum was used as a negative control. The percent cytotoxicity was indicated as average values of triplicate (^*^ *p*<0.01, ^**^ *p*<0.05). Three independent experiments were conducted and similar results were obtained.

It has been reported that mouse ES cells and mesenchymal stem cells are sensitive to serum complement [28], [29]. Complement activity is regulated indirectly by sialic acids on the cell surface [30]. We found that C1 iPS cells did not express sialic acids (Fig. 3A), suggesting that the cells are sensitive to complement. To assess whether C1 iPS cells were susceptible to complement, the cells were incubated with heat–non-treated (complement–non-inactivated) porcine serum. We found that C1 iPS cells released significant amounts of LDH after incubation with the C1 porcine serum, indicating that C1 iPS cells were injured by complement (Fig. 3B).

We examined whether complement hamper the formation of teratomas in vivo. C1 iPS cells were cultured with the porcine serum for 6 hours; then 1×10^6^ cells/site were transplanted into NOG mice (*n* = 6). No teratomas developed in any of the mice at 2 months after transplantation (Table 1) in agreement with a previous report [28]. These results indicate that C1 iPS cells are susceptible to complement-mediated injury.

### Possible control of macrophage phagocytosis by CD47 ligand on C1 iPS cells

**Figure 4:**
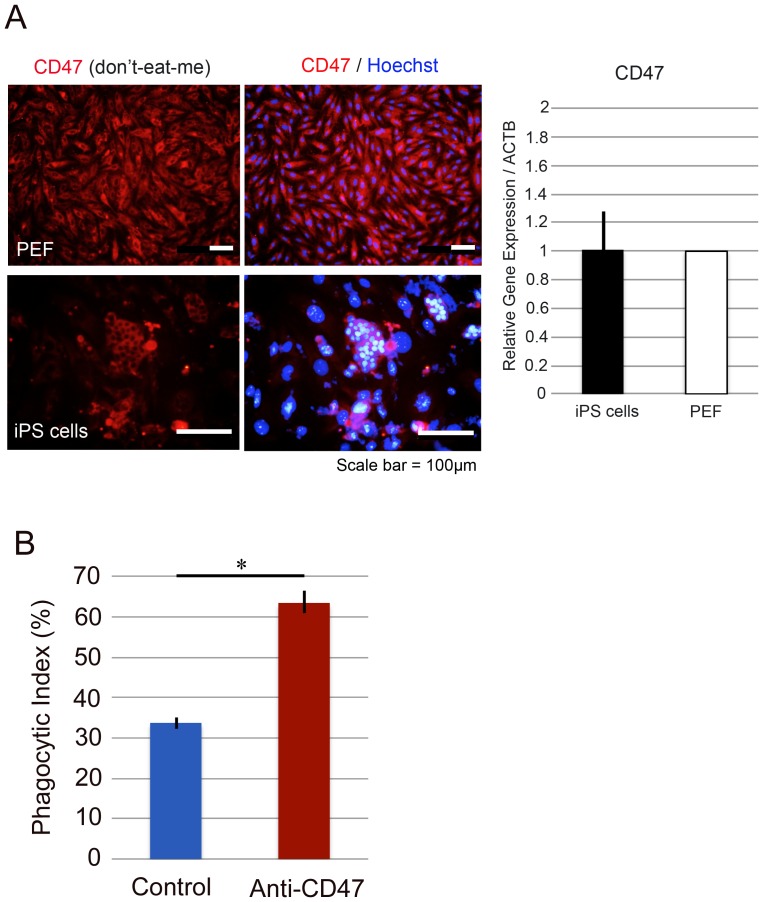
C1 iPS cells evade phagocytosis by macrophages through CD47 ligand. (A) Expression of CD47 was evaluated by immunocytochemistry and semi-quantitative PCR (*n* = 3). C1 iPS cells expressed CD47 to the same extent as fibroblasts. (B) Phagocytosis index (PI) was the number of iPS cells phagocytosed per 100 iPS cells, and it was 33.7±1.5%. The anti-CD47 antibody was then used to block the binding of CD47 to SIRPα. The PI value increased to 63.4±2.8%, showing that CD47 is responsible for the escape of iPS cells from phagocytosis by macrophages (^*^ *p*<0.01).

We examined whether C1 iPS cells are susceptible to macrophage phagocytosis. Macrophage phagocytosis is regulated negatively by interaction of a CD47 ligand (“don't-eat-me” signal) and an immune inhibitory receptor on the macrophage [31], [32], [33], [34]. Semi-quantitative real-time PCR and immunocytochemistry revealed that the C1 iPS cells expressed CD47 to the same extent as fibroblasts (Fig. 4A), suggesting that C1 iPS cells evade macrophage phagocytosis in the C1 recipients.

Therefore, we performed a phagocytosis assay to examine whether C1 iPS cells evade macrophages in vitro. We found that phagocytosis index (PI) was 33.7±1.5%; only one third of iPS cells were engulfed by macrophages (Fig. 4B). On the other hand, significantly more of iPS cells were subjected to phagocytosis (PI = 63.4±2.8%, *p*<0.01) when the cells were incubated with anti-CD47 antibodies (Fig. 4B). These results clearly indicate that engulfment of iPS cells by macrophages is promoted by blocking CD47, suggesting that iPS cells evade macrophagic phagocytosis through the CD47 ligand.

## Discussion

In this study, we investigated immune responses following transplantation of porcine iPS cells into SLA-matched recipients. Our results indicate that SLA-matched iPS cells elicit significantly less cellular and humoral immune responses but they still elicit potent innate immune responses, possibly resulting in the failure in the formation of teratomas. We suggested that innate immunity including serum complement play a role in the in vivo clearance of C1 iPS cells. Several studies have reported that pluripotent stem cells are susceptible to NK cells and serum complement, and that this susceptibility results in the failure to form teratomas in immunodeficient mice [27], [28], [35]. Here, we have shown that it is also the case in the porcine SLA-matched transplantation setting (Table 1).

Our analyses also suggest that macrophages were unlikely to be involved in the rejection of SLA-matched iPS cells. Although C1 iPS cells do not possess SLA class I (Fig. 2A, B) or sialic acids (Fig. 3A), the cells did express CD47 as a marker of self (Fig. 4A). A similar situation occurs in murine red blood cells and in hematopoietic stem cells, which express CD47 that prevents macrophage phagocytosis [31], [33]. The PI values of 33.7±1.5% obtained in this study were low enough to say that C1 iPS cells evade phagocytosis.

The values increased to 63.4±2.8% with the CD47-blocking antibody (Fig. 4B). It is therefore likely that C1 iPS cells evade phagocytosis by macrophages through CD47, also considering other published reports together [32], [34]. For example, PI of pig lymphoblastoid cells with human macrophages was about 25% when CD47 was present, although it was about 65% when CD47 was absent [32]. In the other report, PI of human myeloma cells with mouse macrophages was about 50% when CD47 is present, but it increases to about 70% when CD47 was absent [34].

The T-cell response to porcine iPS cells was attenuated in the SLA-matched allogeneic transplantation setting compared to the SLA-mismatched setting, but still stronger than that to autologous cells (Fig. 1A). There are three possible reasons to explain the remaining cellular and humoral immune responses in the SLA-matched setting. First, unlike strictly matched mouse syngeneic settings, there are a few mismatches in SLA or mitochondrial DNA or other unknown minor antigens between donor iPS cells and recipients [36]. These mismatches may trigger immune responses. In addition, immune reactions can be elicited by sex-mismatches between donors and recipients. Minor histocompatibility antigen in a Y chromosome is immunogenic in a female, although this particular mismatch is unlikely because C1 iPS cells are derived from a female.

Second, the xenogeneic transgenes used to induce pluripotency might induce the immune response to C1 iPS cells. Exogenous “Yamanaka factors” (ectopically expressed *OCT3/4*, *SOX2*, *KLF4* and *c-MYC*) used in the present study are derived from human genes and are thus potentially immunogenic in pigs (Fig. S4 in File S1), although this is not clinically relevant. In addition, a previous study showed that Oct3/4 antigens, even allogeneic ones, are immunogenic and that cells expressing *OCT3/4* become targets of cytotoxic T lymphocytes [37]. C1 iPS cells that retained or reactivated expression of the xenogeneic transgenes might have elicited immunoreactions in the C1 pigs.

Third, STO feeder cells are murine cells and immunogenic in pigs (Fig. 1A). Some STO feeder cells were present in the donor cells (Fig. S5 in File S1), and they may have induced cellular immune responses. Although feeder cells may be excluded with a cell sorter, trypsinizing ES or iPS cells into single cells hampers their ability to develop teratomas [35]. In addition, we transplanted a large number of pig iPS cells (more than 10^7^ in 30–50 dishes at once) into a pig. It is not realistic to prepare such a large number of cells with a cell sorter. In order to completely exclude feeder cells, iPS cells should be expanded under feeder-free conditions. It is certainly an important technique to be developed.

Concerning non-pluripotent stem cells, HLA-matched allogeneic transplantation of hematopoietic stem cells has been successfully conducted for the treatment of hematopoietic malignancies and solid tumors. The cells usually engraft with minimal conditioning of patients [38], [39]. Therefore, it is unlikely that potent natural immunity will occur to hematopoietic stem cells after transplantation. The occurrence of potent immunity seems quite specific to pluripotent stem cells such as iPS cells. It is no wonder, considering that iPS cells lack the expression of MHC class I and sialic acids, eliciting natural immune responses. Other tissue stem cells including hematopoietic stem cells, however, express both of them. That would be a great advantage for clinical applications of iPS cells, because iPS-derived differentiated cells would engraft in recipients but undifferentiated iPS cells contaminated in grafts would be cleared by natural immune responses after transplantation. Whether it is the case is a next step to be examined in the SLA-defined transplantation model.

By extrapolation from our results using the SLA-matched setting, immune responses against undifferentiated human iPS cells will occur in the HLA-matched setting. To our knowledge, this is the first study to assess immune responses to iPS cells in an MHC-matched setting through use of inbred SLA-defined miniature swine.

## Supporting Information

File S1 Contains the following files: **Figure S1.** Characterization of C1 iPS cells. (A) Immunocytochemical staining of C1 iPS cells for Oct3/4, Sox2, Nanog, and Stella. C1 iPS cells were also positive for AP activity. (B) Hematoxylin and eosin staining of teratomas derived from C1 iPS cells showing differentiation into all three germ layers, including stratified squamous epithelia (ectoderm) (a), bundles of nerve fibers (ectoderm) (b), muscle fibers (mesoderm) (c), and columnar epithelia (endoderm) (d). (C) Immunocytochemical staining of differentiated C1 iPS cells in vitro. These cells were positive for GFAP, Tuj-1, Vimentin, FOXA2, AFP, Nestin, Desmin, α-SMA, CK18 or Albumin. **Figure S2.** Transplantation of C1 iPS cells into porcine testes and ovaries. (A) Testes of C1 recipient pigs (a). Injection of C1 iPS cells into the testis using an ultrasound method (b). The yellow ellipse indicates the injection needle (c). (B) Injection of C1 iPS cells into the ovary after laparotomy (a). The transplanted side was labeled using string (b). The ovary on the non-transplanted side (c). (C) Genealogy of C1 Clawn miniature swine used in this study. The donor of the iPS cells was pig AT25. The SLA-matched recipients were pig CT19, CQ38, CU65, SF65 and SD57. Pig CQ74 was used as an SLA-mismatched recipient. The C1 strain was carefully maintained and the C1 status was confirmed by PCR (indicated by *). Pig CQ74 was unexpectedly C2 (indicated by **). The real parent pig P was considered to be C2. **Figure S3.** Serum concentrations of IFN-γ in pigs. Serum concentrations of IFN-γ were measured by ELISA. C1 PBMCs co-cultured with C2 PBMCs were used as a positive control. Serum IFN-γ was undetectable in the SLA-matched C1 recipients, although it was clearly detectable in the SLA-mismatched setting. Two independent experiments were conducted in triplicate and similar results were obtained, one of which was shown here. Each data point represents the mean ± SEM. **Figure S4.** Sustained expression of the transgenes in C1 iPS cells and teratomas. Expression of the exogenous Yamanaka factors was evaluated by RT-PCR. The transgenes were derived from human genes. The primers for RT-PCR were designed to detect the specific retroviral sequences. β-actin was used as a loading control. **Figure S5.** Feeder cells present in donor cells. Flow cytometric analysis showed that feeder cells were present in donor cells. EGFP-labeled C1 iPS cells were used to distinguish from mouse feeder cells (STO). C1 iPS cells were harvested by trypsinization and then incubated on gelatin-coated dishes for 15 min to remove feeder cells. 27.4% of the harvested cells were feeder cells. **Table S1.** Primer sets used in the present study.
(DOC)
