# Supplementary material for: MHC-Matched Induced Pluripotent Stem Cells Can Attenuate Cellular and Humoral Immune Responses but Are Still Susceptible to Innate Immunity in Pigs
Source: PLoS One. 2014 Jun 13;9(6):e98319. doi: 10.1371/journal.pone.0098319 (PMC4057111; doi:10.1371/journal.pone.0098319)
Supplement: File S1 — Contains the following files: Figure S1. Characterization of C1 iPS cells. (A) Immunocytochemical staining of C1 iPS cells for Oct3/4, Sox2, Nanog, and Stella. C1 iPS cells were also positive for AP activity. (B) Hematoxylin and eosin staining of teratomas derived from C1 iPS cells showing differentiation into all three germ layers, including stratified squamous epithelia (ectoderm) (a), bundles of nerve fibers (ectoderm) (b), muscle fibers (mesoderm) (c), and columnar epithelia (endoderm) (d). (C) Immunocytochemical staining of differentiated C1 iPS cells in vitro. These cells were positive for GFAP, Tuj-1, Vimentin, FOXA2, AFP, Nestin, Desmin, α-SMA, CK18 or Albumin. Figure S2. Transplantation of C1 iPS cells into porcine testes and ovaries. (A) Testes of C1 recipient pigs (a). Injection of C1 iPS cells into the testis using an ultrasound method (b). The yellow ellipse indicates the injection needle (c). (B) Injection of C1 iPS cells into the ovary after laparotomy (a). The transplanted side was labeled using string (b). The ovary on the non-transplanted side (c). (C) Genealogy of C1 Clawn miniature swine used in this study. The donor of the iPS cells was pig AT25. The SLA-matched recipients were pig CT19, CQ38, CU65, SF65 and SD57. Pig CQ74 was used as an SLA-mismatched recipient. The C1 strain was carefully maintained and the C1 status was confirmed by PCR (indicated by *). Pig CQ74 was unexpectedly C2 (indicated by **). The real parent pig P was considered to be C2. Figure S3. Serum concentrations of IFN-γ in pigs. Serum concentrations of IFN-γ were measured by ELISA. C1 PBMCs co-cultured with C2 PBMCs were used as a positive control. Serum IFN-γ was undetectable in the SLA-matched C1 recipients, although it was clearly detectable in the SLA-mismatched setting. Two independent experiments were conducted in triplicate and similar results were obtained, one of which was shown here. Each data point represents the mean ± SEM. Figure S4. Sustained expression of the [file pone.0098319.s001.doc]

**Figure S1**


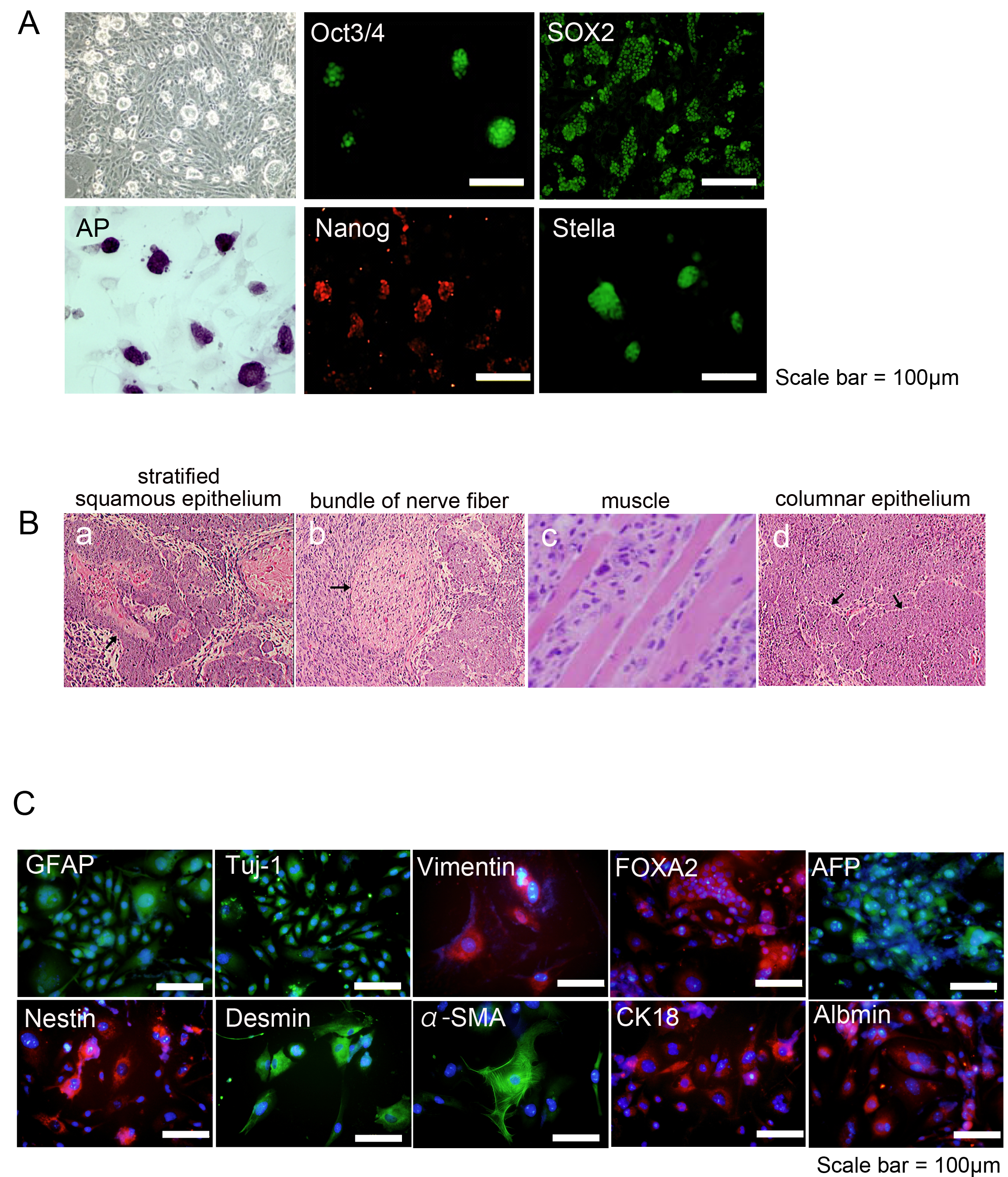


**Figure S2**


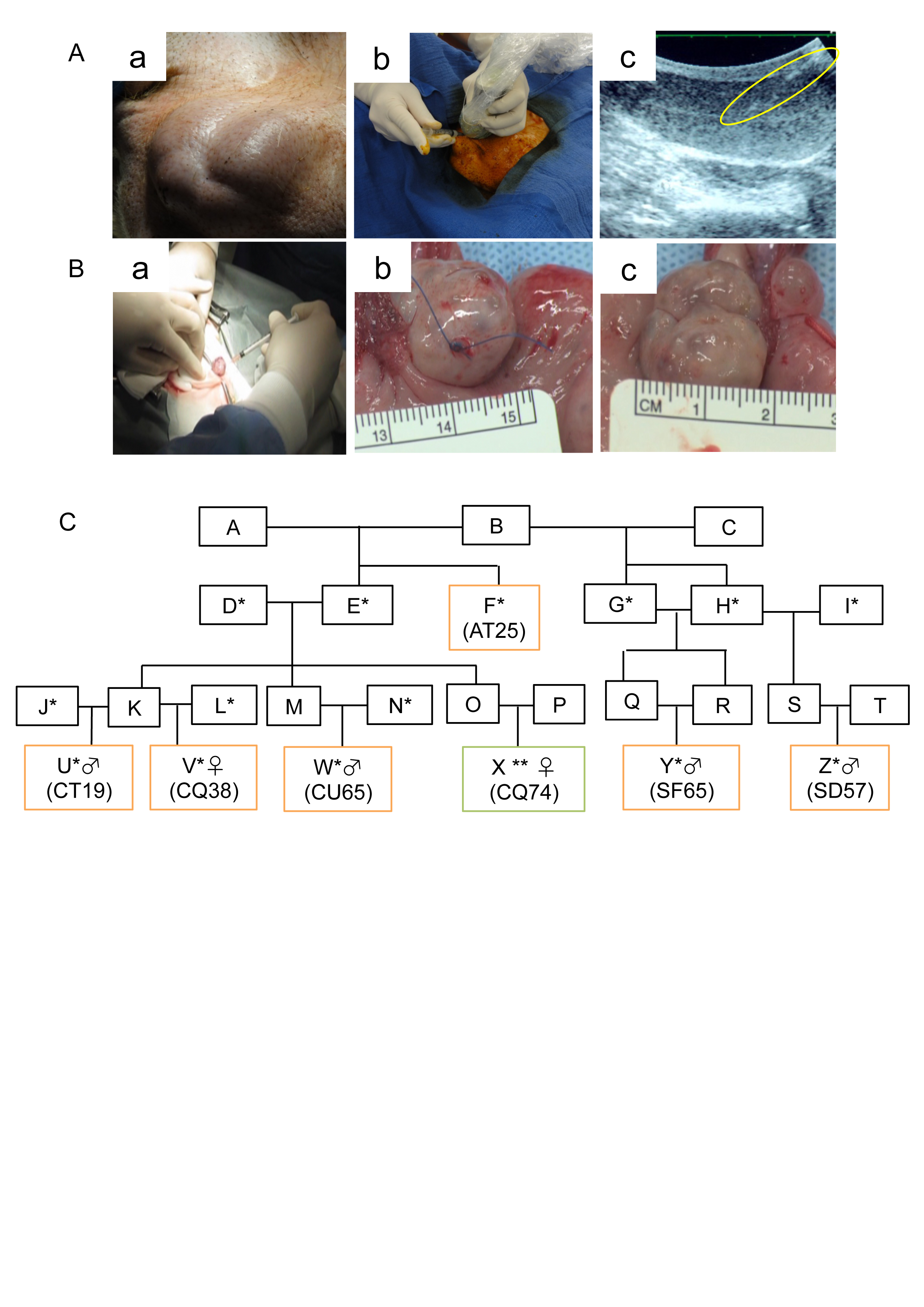


**Figure S3**

**Figure S4**


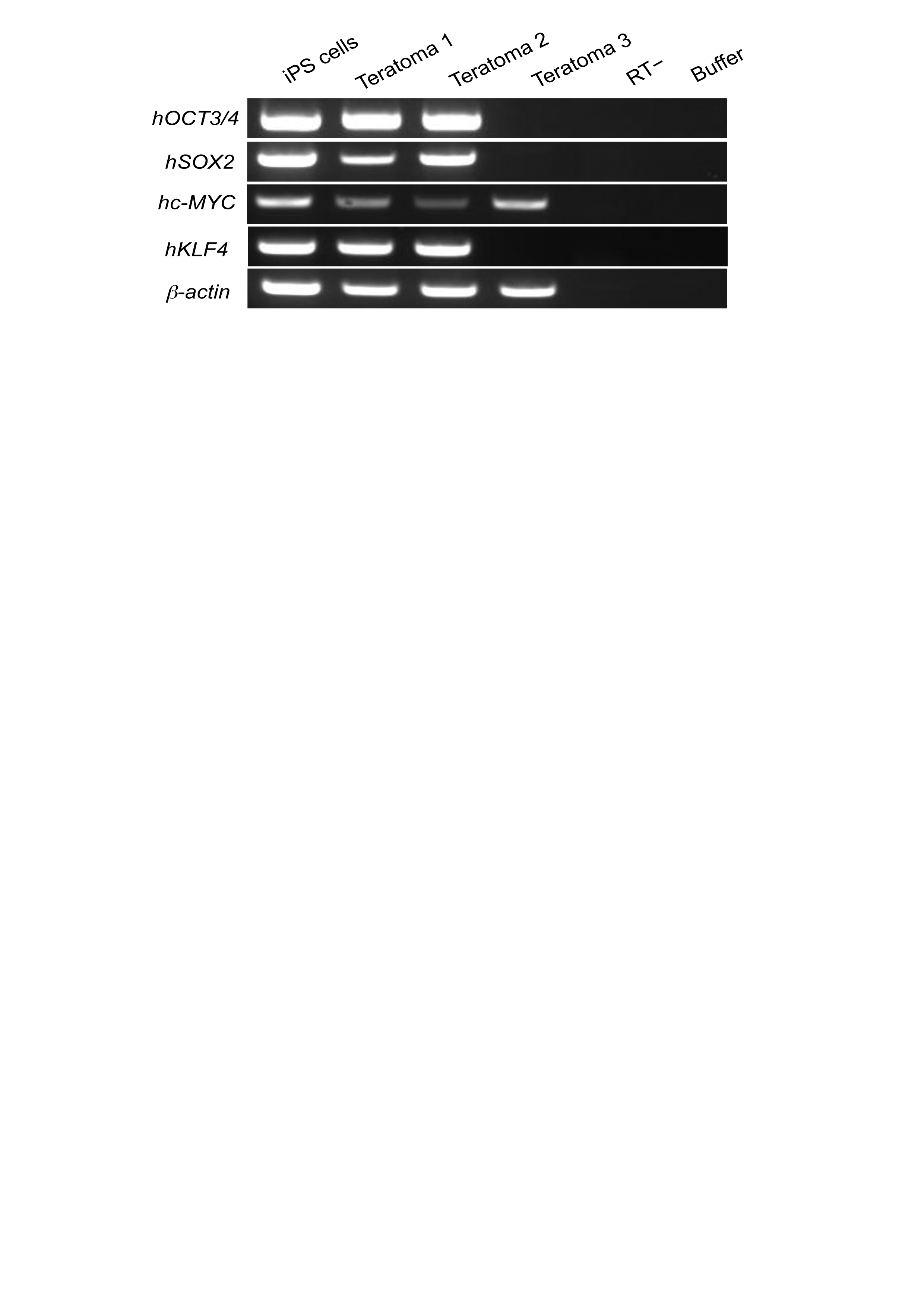


**Figure S5**

**Table S1. Primer sets used in the present study.**

| **Gene** | **Strand** | **Sequence** | **Annealing** |
| --- | --- | --- | --- |
|  |  |  | **Temperature** |
| SLA class I | F | CCTCTTCCTGCTGCTGTCG | 64 |
|  | R | AGCGTGTCCTTCCCCATCT |  |
| ULBP1 | F | GGCCTGCGATACTCACTCTC | 63 |
|  | R | AGGAAGTCCCCAACGTCTTT |  |
| MICA | F | GTCGTTGCACAGCACAATCT | 63 |
|  | R | TACGCCATGTCTCAGTGCTC |  |
| GAPDH | F | ACCTGCCGCCTGGAGAAACC | 63 |
|  | R | GACCATGAGGTCCACCACCCTG |  |
| CD47 | F | TCAGATCCTGGGGTTTCTTG | 63 |
|  | R | GTAGGCAGTTCCCAACCAAA |  |
| β-actin | F | CATCACCATCGGCAACGA | 62 |
| (qRT-PCR) | R | GTTGGCGTAGAGGTCCTTCCT |  |
| hOCT3/4-S944 | F | CCCCAGGGCCCCATTTTGGTACC | 53 |
| hSOX2-S691 | F | GGCACCCCTGGCATGGCTCTTGGCTC | 53 |
| hKLF4-S1128 | F | ACGATCGTGGCCCCGGAAAAGGACC | 53 |
| hMYCS1011 | F | CAACAACCGAAAATGCACCAGCCCCAG | 53 |
| pMXs-L3205 | R | CCCTTTTTCTGGAGACTAAATAAA |  |
